# Systematic Review and Meta-Analysis of Surgical Zipper Technique versus Intracutaneous Sutures for the Closing of Surgical Incision

**DOI:** 10.1371/journal.pone.0162471

**Published:** 2016-09-09

**Authors:** Dezhi Chen, Jian Song, Yong Zhao, Xun Zheng, Aixi Yu

**Affiliations:** Department of Orthopedics, Zhongnan Hospital of Wuhan University, Wuhan, Hubei, 430071, P.R. China; University Medical Center of Princeton at Plainsboro, UNITED STATES

## Abstract

**Background:**

It is controversial whether surgical zipper technique (SZT), a non-invasive method of surgical wound closure, achieves a better outcome of incision healing than intracutaneous sutures (IS) in the surgery. This meta-analysis was performed to systematically analyze whether surgical zipper is superior to suture material for the incision closure.

**Methods:**

Databases and reference lists were searched for randomized controlled trials (RCTs) comparing SZT with IS for the incision closure.

**Results:**

Four RCTs with 678 patients were identified and analyzed. Compared with IS, SZT achieved similar incidence of postoperative complications, less time for incision closure, less cost of both surgeons’ time and operating room time, no need for removing sutures and more comfort for the patients. Besides, SZT achieved perfect aesthetic results in various types of incisions with the exception of those with substantial curvatures, those with secretions, in obese patients or those under high tension.

**Conclusion:**

The non-invasive zipper technique may be a more attractive option of incision closure in a wide spectrum of surgical areas.

## Introduction

The healing of a skin wound is a complex process requiring the collaborative efforts of many tissues and cell lineages [1]. The goal of incision healing is to prevent wound infection and achieve a good cosmetic result. To date, various types of incision closure techniques have been applied to clinical practice, including synthetic sutures, absorbable sutures, staples and adhesive compounds [2–4]. It is known that in wound closure, intracutaneous suture technology often gives a good cosmetic result. However, traditional suture material may create tensions across the wound edges after contributing to inadequate circulation and supporting the bacterial adhesion to surgical sutures with a potential risk of postoperative infection [5,6], which would disturb the natural healing process of incision.

Recently, a new closure technique named surgical zipper technique (SZT) is being widely used to facilitate the incision healing [7–9]. It is a combination of microporous polyester and a zipper that is coated with acrylate adhesive, which could provide uniform force on the wound edge to facilitate a natural healing process of the incision [7–9]. In the process of incision closure, the underlying fascia is closed in a standard manner using absorbable suture material to close the subcutaneous tissue for the reduction of skin tension and then the skin wound is closed using the surgical zipper [1,10–12]. The zipper can be used for straight or slightly curved incisions [8]. The application of the zipper should be 2–4 cm longer than the wound and leave a distance of 0.5 cm between the zipper teeth and the edge of the incision. The incision should be elongated manually to approximate the wound edges. The operator should close the zipper at the same time when pulling gently on the rear loop. The zipper is available in seven sizes: 6, 12, 20, 25, 30, 40, and 50 cm [8] and it can be used for a wound of 4 to 47 cm in length. Surgical zipper is also designed for early incision inspection just by opening it and then closing it. But in most cases, the zipper was not routinely opened until removal [1, 10, 11].

Several published randomized controlled trials (RCTs) have compared SZT with IS in incision closure. However, the conclusions are not clear and systematic. The purpose of this study is to systematically review the efficacy of the non-invasive zipper technique versus intracutaneous method for incision healing.

## Methods

The study protocol was shown in S1 PRISMA Checklist (2009).

### Search strategy

RCTs comparing SZT with IS for the efficacy of incision healing were identified and retrieved from Medline, Embase, Cochrane Library database and Chinese Biological Medline using the free text terms “Medizip” “zipper” “sutur*” in all fields in combination with the Boolean operators AND or OR. We systematically searched these databases from their establishment to March 2016. The reference lists were checked for additional studies.

### Selection criteria

All abstracts identified by the search strategy were screened manually and full-text articles were then reviewed for closer examination. Studies were included if: 1) The study was a RCT (the article mentioned using randomization). 2) The deeper structures in the wound were closed using absorbable suture material and then the skin wound was closed in both groups by IS and SZT, respectively. 3) The study reported detailed outcomes of SZT versus IS. 4) Studies containing two or more comparative arms, one of which met the above conditions, were included in the study.

### Data extraction

Data was independently extracted by two investigators from the articles and checked by other authors. Discrepancies were resolved by consensus discussions.

General study information, baseline characteristics of the patients, types of surgeries, details of the process of surgeries and postoperative management would be extracted from each eligible study. Data for the mean value and standard deviation in the studies would be converted according to formulas because of different units of measurement. All data was extracted from the studies using a data extraction form.

### Assessment of risk of bias

We systematically assessed the risk of bias according to the guidelines of the Cochrane collaboration [13]. Six terms have been considered relevant including random sequence generation, allocation concealment, baseline characteristic, eligibility criteria, description of loss to follow-up and drop-out, intention-to-treat analysis. Studies with one or two negative answers were regarded at a moderate risk of bias and studies with three or more negative answers were qualified as high risk of bias.

### Statistical analysis

Statistical studies were conducted using software Revman 5.3 [14]. Pooled risk ratio (RR) with 95% confidence interval (CI) was calculated for the incidence of postoperative complication including total wound infection (TWI), superficial wound infection (SWI) and blister, and mean difference (MD) was calculated for outcomes that were continuous variables such as the time for incision closure. All pooled outcome measures were determined using random-effects models as described by DerSimonian and Laird [15]. A *p* value of < 0.05 was considered statistically significant.

The *Q* statistic and *I*^*2*^ value were used to assess heterogeneity of treatment effect. A *p* value of < 0.05 and *I*^*2*^ value of >50% were used to indicate the presence of significant heterogeneity. We conducted the sensitivity analysis by excluding each individual study and recalculating the summary RR and CI to evaluate whether the results were affected significantly. Otherwise, we presented the narrative syntheses when the data of outcome was inappropriate to combine but important to analyze.

## Results

### Description of eligible trials

**Fig 1:**
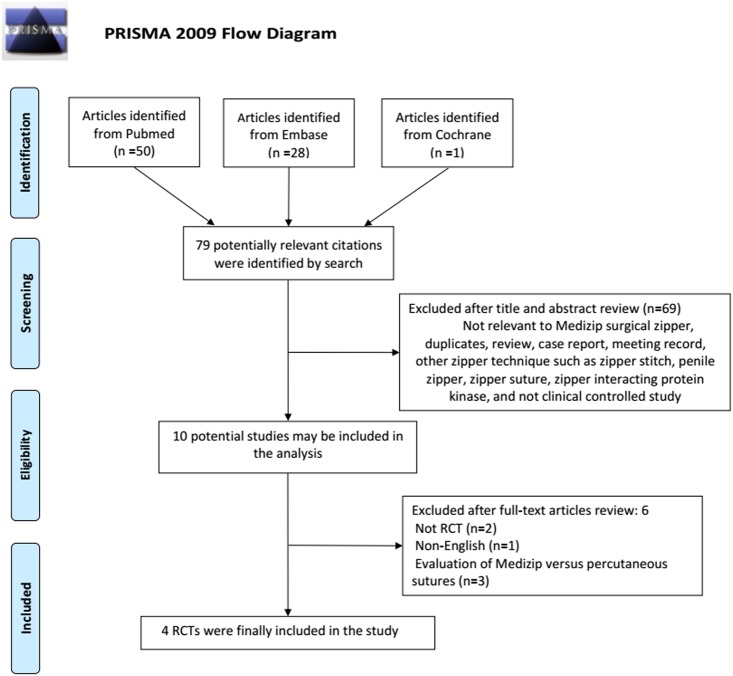
Flow diagram for study selection.

Fig 1 shows the flow chart of the studies retrieved and excluded. We identified 79 potentially relevant articles from the databases. Sixty-nine articles were excluded after screening their titles and abstracts, and the rest was analyzed by reading the full text. Finally, 4 RCTs were deemed eligible and included in this study.

Table 1 summarises the characteristics of included RCTs. Four trials involving 678 patients met the inclusion criteria. Of them, 333 patients were randomly assigned to the SZT group and 345 patients assigned to the IS group. Two trials [10, 11] were conducted by the same group and published in the same year.

**Table 1:** Characteristics of included randomized controlled trials in the meta-analysis.

Study, year	Country	Study period	Number of patients		Mean age			Mean BMI			Sex M/F	
			SZT	IS	SZT	IS	P value	SZT	IS	P value	Men	Women
Risnes [10], 2002	Norway	June 2000- July 2001	150	150	66.3	63.1	0.05	-	-	-	204	96
Roolker [1], 2001	Netherlands	November 1996- February 1998	60	60	44.9	49.1	0.19	-	-	-	45	75
Xu [12], 2014	China	July 2011- June 2012	45	45	13.2	13.5	NS	17.8	17.2	0.72	-	-
Risnes [11], 2002	Norway	October 1999- July 2001	78	90	-	-	-	-	-	-	-	-

NS, not significant;—, data not available; SZT, surgical zipper technology; IS, intracutaneous sutures; BMI, body mass index

Table 2 summaries the details of surgeries and postoperative management. The curve magnitude of the incisions was reported in only one trial [12] which made incisions with substantial curvature beyond 45 degrees.

**Table 2:** Details of the surgeries and postoperative management in the four RCTs.

Study, year	Type of surgery	Suture material	Length of incision	Mean curve magnitude	Time for removal of the zipper
Risnes [10], 2002	Open-heart surgery (94 coronary artery bypass grafting, 66 valve replacement or plasty, 58 combined coronary artery bypass grafting and valve replacement and 82 various other procedures)	Poliglecaprone (Monocryl 3–0) intracutaneous suture	-	-	The zipper was routinely not opened until removal after 12 days.
Roolker [1], 2001	Orthopaedic surgery (20 knee operations, 20 hip operations, 20 spine operations)	PDS suture	Mean length of SZT group, 20.5 cm; mean length of IS group, 18.2 cm; p value, 0.13	-	The zipper was not opened but removed between 10 and 14 days after the operation.
Xu [12], 2014	Posterior spinal fusion surgery	4–0 subcuticular Monocryl sutures	Mean length, 31.5 cm, range from 29.2 cm to 34.2 cm	52.3°, ranging from 45°to 70°	The surgical zipper was removed on the seventh day after operation.
Risnes [11], 2002	Saphenous vein harvesting	Monocryl 3–0 poliglecaprone intracutaneous suture	Range from 2 cm to 46 cm	-	The zipper was routinely not opened until removal after 12 days.

SZT, surgical zipper technique; IS, intracutaneous sutures; -, not reported in the article

### Quality of eligible trials

There was a good agreement among the reviewers about the selection criteria and quality assessment of the studies. Table 3 lists assessment of the quality for the four included trials.

**Table 3:** Assessment of the risk of bias for the four trials.

Study, year	Randomization method	Allocation concealment	Homogeneous Baseline characteristic	Eligibility criteria	Loss to follow-up and drop-out described	Intention-To-Treat analysis	Score
Risnes [10], 2002	Envelope method with blocks of 40 patients	Envelopes	No	Yes	Yes	No	Moderate risk
Roolker [1], 2001	Unclear	Unclear	Yes	Yes	No participant absent	Not needed	Moderate risk
Xu [12], 2014	Unclear	Sealed identical opaque envelopes	Yes	Yes	No participant absent	Not needed	Moderate risk
Risnes [11], 2002	Block number	Unclear	Yes	Yes	Yes	No	Moderate risk

In the four studies, only two studies [10, 11] specified the randomization method of block randomization and two trials [10, 12] applied adequate allocation concealment by using Envelopes. Baseline characteristics were similar in the three trials [1, 11, 12]. All four RCTs revealed the eligibility criteria for patients enrolled in. Two studies [10, 11] specified numbers of patients loss to follow-up and drop-out but no intention-to-treat (ITT) analysis was performed in both trials. Blinding was not applied in these trials because of the nature of the intervention. Of these studies, all four trials were regarded to have a moderate risk of bias.

### Postoperative complication

**Fig 2:**
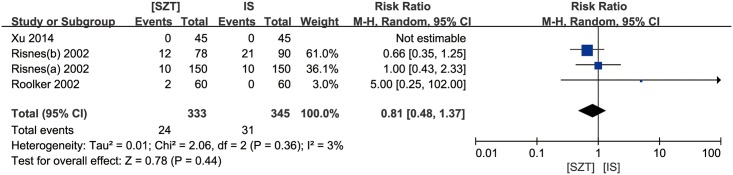
Forest plot for TWI between SZT and IS. Risk ratios are shown with 95 per cent confidence intervals.

All four trials reported data for the total wound infection and the results indicated no significant statistical heterogeneity between the trials (*p* = 0.36). Compared with IS, SZ did not increase the incidence of postoperative incision infection (pooled RR 0.81, 95% CI, 0.48 to 1.37, *p* = 0.44) (Fig 2).

**Fig 3:**
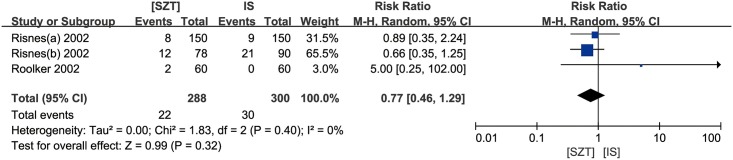
Forest plot for SWI between SZT and IS. Risk ratios are shown with 95 per cent confidence intervals.

**Fig 4:**
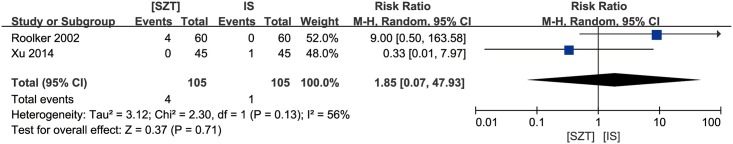
Forest plot for blister between SZT and IS. Risk ratios are shown with 95 per cent confidence intervals.

The incidence of SWI and blister was reported respectively in three and two studies. The results indicated that, compared with IS, SZT showed similar incidence of SWI (pooled RR 0.77, 95% CI, 0.46 to 1.29, *p* = 0.32) (Fig 3) and blister (pooled RR 1.85, 95% CI, 0.07 to 47.93, *p* = 0.71) (Fig 4). There was also no significant difference in the incidence of deep wound infection (DWI) [1, 10], dehiscence of the wound [1, 12] and wound separation [1].

### The time for incision closure

**Fig 5:**
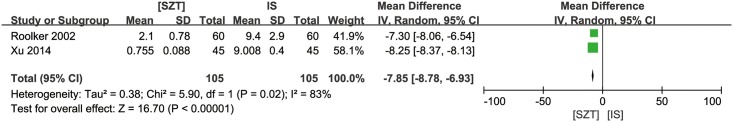
Forest plot for incision closure time between SZT and IS. Weighted mean differences are shown with 95 per cent confidence intervals.

Two trials [1, 12] involving 210 patients reported the time for surgical wound closure. In the random-effects model, the application of zipper technique significantly decreased the time consumed for incision closure compared with that of intracutaneous closure (MD -7.85, 95%CI, -8.78 to -6.93, *p*<0.00001). Heterogeneity of treatment effects was significant (*p* = 0.02) (Fig 5).

### Cosmetic outcome

The cosmetic result was evaluated by patients themselves with different scales at the follow-up visit in these studies. It showed that SZT achieved better aesthetic results in the patients 6 weeks after the closing of sternal wound [10] and 6 weeks after leg wound closure [11], but showed no significant difference 6 weeks after the orthopaedic surgery [1] and one year after posterior spinal fusion surgery [12].

### Sensitivity analysis

Our sensitivity analysis performed by removing each individual study from the above analyses did not markedly change the RRs or 95% CIs, indicating that the conclusions were confirmed.

## Discussion

The results demonstrate that SZT significantly decreased the time used for incision closure, the cost of both surgeons’ time and operating room time, and the level of discomfort in the patients undergoing incision closure, and had similar incidence of postoperative complications and similar or even better cosmetic results.

Skin infection is a common complication occurring after surgical incision closure. The patients’ own skin is considered to represent the main source of pathogenic bacteria. Traditional wound closure techniques inevitably leave needle tracks and give micro-organisms additional routes of entering the incisions through suture material at the time of operation and suturing incisions. It may represent an important factor in the development of postoperative incision infection [5, 16]. Using the non-invasive closure system, the risk of infection from skin pathogens is considered to be reduced due to the absence of perforation of potentially infected structures and tension in the wound area [17–22]. The zipper technique may be more effective in decreasing the potential risk of postoperative wound infection although practical results show similar complication rate between the two techniques.

Acquiring good cosmetic results is a common demand of the patients undergoing surgical procedures. The most important requirements for good scar result are primary wound healing without tension and lack of trauma to the wound edges [19, 20]. SZT provides a homogeneous distribution of tension across the entire incision region, and causes less edema than IS [20, 21], which provides adequate circulation for incision healing. However, the result shows that SZT has no significant advantages of aesthetic result in orthopaedic surgery [1] and posterior spinal fusion surgery [12]. It may be explained for the incisions with substantial curvature of more than 20 degrees in the two surgeries, which is an important limitation to the use of SZT [23]. Other limitations of the zipper are the lack of application in closure of incisions with high tension or secretions, and incisions in obese patients [23]. The use of the zipper is of especially great value in paediatric and young oncology patients [8].

One of the most important advantages of SZT is the significantly less time consumed in the wound closure, contributing to less cost of both surgeons’ time and the operating room time [1, 12]. Roolker et.al [1] reported that the cost for using the zipper was $ 13 on average and for the intracutaneous sutures, $ 8. There was a significant difference in surgeon’s time cost related to the time of closure: zipper versus intracutaneous was $ 2 vs $ 11.6. The cost of operation room time differs from hospital to hospital and is therefore not included. So we could conclude SZT potentially saves a substantial amount of medical resources, thus reducing the financial burden on the health care system [1, 12].

SZT represents an easy and painless application process of apposing skin incision as well as an easy and painless removal process, which decreases the level of discomfort for patients undergoing surgery. Skin perforation and needle stick injuries can be avoided, trauma to the wound edge minimized and wound edge approximation is homogeneous, thus promoting a natural, tension-free healing process. After the surgery, the zipper allows an uncomplicated skin wound inspection and offers no need for removing sutures and bandages. Surgical zipper can keep its adhesive properties for at least 10 days and routinely not be opened until removal in most cases [1, 10, 11]. Its removal is painless for the patients [12]. Furthermore, the fenestration in the zipper strips allows good aeration of wound and prevents the accumulation of sweat which may lead to the sogginess of wound [9].

In the analysis, the following limitations should be considered. First, the sample size was relative small, which may decrease the level of evidence. Second, all included RCTs were not well designed. Though all trials mentioned randomization, many studies did not report the randomization method, concealment of allocation. Third, heterogeneity was obvious with respect to incision closure time. It might be caused by different kinds of surgeries in patients of different age groups, difference in surgeon skill. Furthermore, postoperative cosmetic results were evaluated with different scales and we could not pool the related data to gain a more powerful conclusion. Finally, language bias should be considered. Though we applied search strategies of no language restriction, all trials were collected by language limitation to Chinese and English, thus may weaken the applicability of the conclusions.

In conclusion, SZT may be a preferable option in the closing of surgical incision for the ease and speed of incision, cost-effective, no need for removing sutures and bandages, similar incidence of postoperative complication, less discomfort in the patients and better cosmetic results in a wide spectrum of surgical areas.

## Supporting Information

S1 PRISMA Checklist PRISMA 2009 checklist. (DOC)
